# The Geographic Distribution of Liver Cancer in Canada Does Not Associate with Cyanobacterial Toxin Exposure

**DOI:** 10.3390/ijerph121214969

**Published:** 2015-11-30

**Authors:** Meaghan A. Labine, Chris Green, Giselle Mak, Lin Xue, Janet Nowatzki, Jane Griffith, Gerald Y. Minuk

**Affiliations:** 1 Department of Pharmacology and Therapeutics, University of Manitoba, Winnipeg, MB R3T 2N2, Canada; meaghanlabine@hotmail.com; 2 Cancer Care Manitoba, University of Manitoba, Winnipeg, MB R3T 2N2, Canada; greenc@cc.umanitoba.ca (C.G.); giselle.mak@cancercare.mb.ca (G.M.); lin.xue@cancercare.mb.ca (L.X.); janet.nowatzki@cancercare.mb.ca (J.N.); jane.griffith@cancercare.mb.ca (J.G.); 3 Morberg Family Chair in Hepatology, Department of Internal Medicine, University of Manitoba, John Buhler Research Centre, 715 McDermot Ave. Winnipeg, MB R3E 3P4, Canada

**Keywords:** cyanobacteria, cyanotoxins, microcystin, liver cancer, blue-green algae, eutrophication, hepatitis B, hepatitis C

## Abstract

*Background*: The incidence of liver cancer has been increasing in Canada over the past decade, as has cyanobacterial contamination of Canadian freshwater lakes and drinking water sources. Cyanotoxins released by cyanobacteria have been implicated in the pathogenesis of liver cancer. *Objective*: To determine whether a geographic association exists between liver cancer and surrogate markers of cyanobacterial contamination of freshwater lakes in Canada. *Methods*: A negative binomial regression model was employed based on previously identified risk factors for liver cancer. *Results*: No association existed between the geographic distribution of liver cancer and surrogate markers of cyanobacterial contamination. As predicted, significant associations existed in areas with a high prevalence of hepatitis B virus infection, large immigrant populations and urban residences. *Discussion and Conclusions*: The results of this study suggest that cyanobacterial contamination of freshwater lakes does not play an important role in the increasing incidence of liver cancer in Canada.

## 1. Introduction

Rates of primary liver cancers, of which hepatocellular carcinoma is the most common, have been increasing in low-incidence countries such as Canada [1]. Canadian liver cancer incidence rates are 3.4% per year in males and 2.2% per year in females [1,2]. In Canadian women, the incidence of liver cancer is increasing more so than all other cancers, and is second only to thyroid cancer in Canadian men [2,3].

While increases in injection drug use and immigration of individuals from countries endemic for viral hepatitis B and C have almost certainly contributed to the increase in liver cancer, an uneven geographical distribution of liver cancer cases would be in keeping with exposure to certain environmental factors that possess direct or indirect carcinogenic properties [2,4,5,6,7,8]. Previously described liver cancer risk factors include but are not limited to hepatitis B or C viral infections, alcohol abuse, aflatoxin exposure, recent immigration and urban rather than rural residences [9,10,11,12,13].

Cyanobacteria, otherwise known as blue-green algae, are a diverse group of photosynthetic prokaryota that are ubiquitous to all freshwater ecosystems. Certain species of cyanobacteria can produce toxic secondary metabolites (cyanotoxins) which are harmful to humans and animals [14,15,16]. Cyanotoxin accumulation in surface waters is associated with excessive cyanobacterial growth, characterized by biomass accrual and bloom formation. Within temperate climates, eutrophication (low nitrogen-to-phosphorous ratios) of inland surface waters, driven largely by nutrient rich effluent (urbanization, agriculture and farming), is a key factor contributing to bloom formation [17,18,19,20].

Microcystins (MCs) are a group of hepatotoxins commonly produced by freshwater cyanobacteria [21,22,23]. MCs pose a threat to drinking water quality due to their environmental resilience, bioaccumulation and toxicity [16,24,25]. *Microcystis aeruginosa* is a toxigenic species of freshwater cyanobacteria, capable of releasing microcystin-LR (MC-LR). Although bloom formation is not a direct measure of environmental MC concentration, there is an association between *Microcystis aeruginosa* abundance and aqueous MC-LR concentration [18]. Concentrations of MC in a small number (*N* = 5) of freshwater lakes within the Canadian province of Manitoba range from 0.2–205 µg/L (unpublished data from Manitoba Water Stewardship). Acute human exposure to MCs is associated with nausea, vomiting, gastroenteritis and severe hemorrhagic hepatitis [25]. More long-term *in vitro* and *in vivo* studies describe MCs as being both direct and indirect liver carcinogens, however the precise mechanism(s) has yet to be identified [26,27,28,29,30,31,32].

The long-term health effects associated with MC exposure in humans is not fully understood. Epidemiological studies conducted in China, Serbia and the United States describe positive associations between chronic exposure to MC contaminated drinking water and the incidence of liver cancer [33,34,35,36]. Moreover, in China it was observed that liver cancer rates decreased following the introduction of cyanotoxin-free drinking water in the affected communities [33,34].

In Canada, toxigenic cyanoblooms have been observed in numerous freshwater bodies including: Lake Winnipeg (Manitoba), Lake Erie (Ontario) and Lake Champlain (Quebec) [37,38,39,40]. Despite the increased prevalence of cyanoblooms in Canada, drinking water monitoring programs are inconsistent across the country [21]. Thus to obtain insight into the extent of cyanobloom formation and potential MC exposure, “up-stream” activities producing nutrient rich effluent (urbanization, agricultural and farming activity), have been employed as surrogate markers of cyanobacterial contamination [19,20].

Within Canada, the impact of MC exposure on population health is unknown. In the present study, we documented the geographical distribution of liver cancer relative to the presence of surrogate markers for cyanobacterial exposure, to determine whether an association exists between the two.

## 2. Methodology

### 2.1. Data Sources

Liver cancer incidence data for the years 1996 to 2004 were obtained from the National Cancer Registry for Canada. These data were received in a highly aggregated form, with no age or gender breakdown, and case counts provided at the Census Division (CD) level. Population data were also supplied by the National Cancer Registry for the years 1996 to 2004 at the CD level. In Canada there are 284 CDs with a mean population of 108,304, a median population of 40,355, and a range of 1397 to 2.5 million (1996–2004 average). For the 92 CDs in which the liver cancer case count was less than 5, case counts were suppressed by the National Cancer Registry in order to protect confidentiality. Case counts for suppressed CDs were imputed in this study by calculating the expected number of cases. This was accomplished by applying national liver cancer rates for 1996 to 2004 to the age and gender specific population counts in each suppressed CD. Expected case counts were assumed to be a reasonable estimate if they were less than 5, since the actual case counts must be less than 5. Similarly, if the expected case count was greater than 5, knowing that actual case counts were less than 5 for suppressed CDs, a reasonable estimate was assumed to be 4. Since it was not possible to directly calculate age and gender standardized rates for this study (age and gender were not supplied with the case data), all analyses were restricted to using the 40+ population as a denominator in order to partially control for the effect of age on study results. Over 95% of liver cancer cases occur in the 40+ population [41].

Surrogate markers of potential cyanobacterial exposure included: agricultural activity (percent of land devoted to agriculture), cattle and swine densities. Established risk factors for liver cancer included population based estimates of age, gender, incidence of hepatitis B and C, alcohol abuse, recent immigrant status and urban residence. The variables analyzed in the study, their sources, and methods of calculation are summarized in Table 1.

**Table 1:** Data Sources.

Variable	Source	Method of Calculation
Land Area devoted to Agriculture (%)	2006 Canadian Census of Agriculture (accessed through the Data Liberation Initiative, University of Manitoba)	The reported area of land devoted to agriculture was divided by the total land area of each CD
Cattle Densities (no. of cattle/1000 sq. km)	2006 Canadian Census of Agriculture (accessed through the Data Liberation Initiative, University of Manitoba)	The reported number of cattle in each CD was divided by the land area of each CD.
Swine Densities (no. of swine/100 sq. km)	2006 Canadian Census of Agriculture (accessed through the Data Liberation Initiative, University of Manitoba)	The reported number of swine in each CD was divided by the land area of each CD
Population 40+ (%), 1996–2004	Population Data supplied by National Cancer Registry	No. of persons aged 40 plus divided by the total population, using population denominator data from 1996–2004 combined
Male Population (%), 2001	2001 Canadian Census (accessed through the Data Liberation Initiative, University of Manitoba) http://www12.statcan.ca/english/census01/home/index.cfm	No. of males divided by the total Population
HepB Incidence, cases/100,000, 1989–2004	Notifiable Diseases On-Line http://dsol-smed.phac-spc.gc.ca/dsolsmed/ndis/index_e.html	Reported HepB incidence for the years 1989-2004 were averaged at the provincial level. The average provincial rate was then applied to CDs by province.
HepC Incidence, cases/100 000, 1989–2004	Notifiable Diseases On-Line http://dsol-smed.phac-spc.gc.ca/dsolsmed/ndis/index_e.html	Reported HepC incidence for the years 1989–2004 were averaged at the provincial level. The average provincial rate was then applied to CDs by province.
Alcohol Abuse (%), 40–64 yr olds, 2007	2007 Canadian Community Health Survey http://secure.cihi.ca/indicators/2007/en/datatables_maps07_e.html	Numerator (no. of excessive drinkers 40–64 years of age) and denominator (no. of persons 40–64 years of age) by Regional Health Authority Area were re-scaled to Census Division using the areal interpolation extension in Arc-GIS
Recent Immigrantion (%), 2001	2001 Canadian Census (accessed through the Data Liberation Initiative, University of Manitoba) http://www12.statcan.ca/english/census01/home/index.cfm	Derived directly from reported Census values
Urban/rural	2001 DMTI Population Counts by City (accessed through the Data Liberation Initiative, University of Manitoba) http://www.dmtispatial.com/	CD were classified as Urban if they contained at least one City with a population of 50,000 or more in 2001

### 2.2. Statistical Methods

To visualize spatial trends in liver cancer rates and its predictors, thematic maps were generated using ArcGIS 9.3 [42]. Crude incidence rates for the 40+ population were calculated for 1996 to 2004 combined and used for mapping. Smoothed rate estimates were generated using the Bayesian smoothing function in the GeoDa software [43]. The GeoDa software was set to access data from the five nearest CDs for smoothing. The spatial scan statistic implemented using the Satcan 8 software [44], was used to confirm that the spatial patterns in liver cancer incidence identified in the visualization stage were real and not due to random spatial variation. The software was set to find high rate clusters containing a maximum of 50% of the study population and low rate clusters containing a maximum of 1% of the study population. Detected clusters were tested for significance using 999 Monte Carlo random simulations, with only clusters significant at the *p* < 0.05 level retained for mapping.

The Gini Coefficient was calculated using the EPIDAT 3.0 software package [45], to describe the degree to which liver cancer cases were distributed equally in relationship to the population at risk. The Gini coefficient was calculated from the ranked cumulative proportion of liver cancer cases and the population at risk across CDs. The ecological relationships between liver cancer and its predictors were assessed through the development of a partially adjusted (adjusting only for age and gender at the ecological level) negative binomial regression model using SAS 9.13 [46]. The negative binomial distribution was used to proactively deal with potential over-dispersion in the data. In these models, the count variable was incident liver cancer cases from 1996 to 2004 and the off-set variable was the population for the years 1996 to 2004 combined. Predictor variables were classified into tertiles across geographic areas using the Jenks natural breaks algorithm in ArcGis 9.3 and entered into the regression model as categorical predictors. Model outputs were expressed as rate ratios, using the lowest rate category as the reference where possible.

## 3. Results

**Figure 1:**
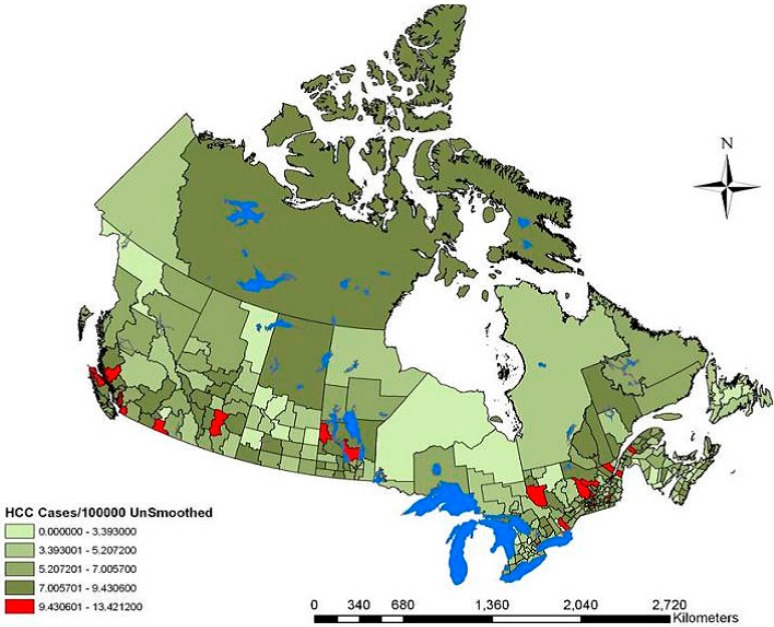
Canadian liver cancer cases per 100,000 within the 40+ population measured between 1996 and 2004. The map depicts the un-smoothed geographic distribution of cases.

**Figure 2:**
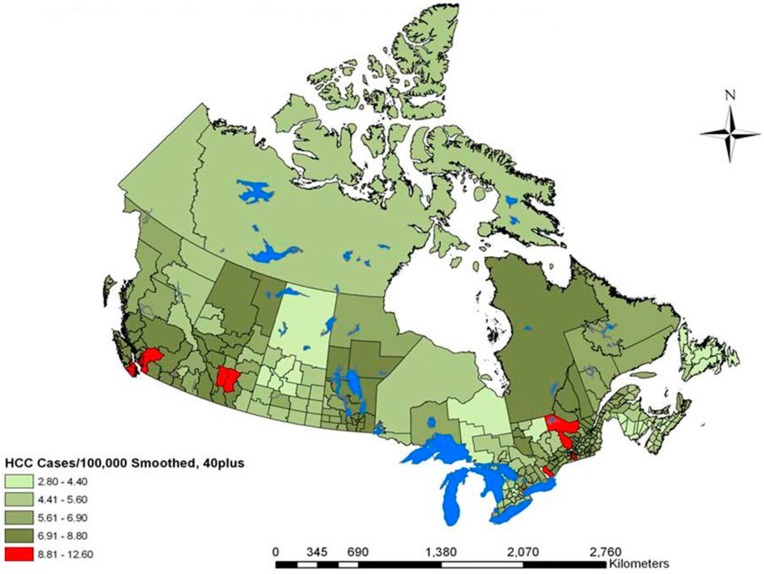
Canadian liver cancer cases per 100,000 within the 40+ population measured between 1996 and 2004. The map depicts the smoothed geographic distribution of cases using the five nearest spatial empirical bayes (five nearest neighbors).

Between 1996 and 2004, there were 9288 incident cases of liver cancer in the 40+ population of Canada, with an annual crude rate of 7.55 cases/100,000 person years. Rates ranged from 2.8 to 12.6 cases/100,000 person years. The thematic map of unsmoothed liver cancer rates (Figure 1) revealed that the highest rates of liver cancer were in the urban centers of Vancouver, Calgary, Montreal and Toronto. These centers also persisted as high rate sites in the smoothed liver cancer map (Figure 2). The lowest rates of liver cancer were observed in northern Saskatchewan, parts of British Columbia, Ontario, Maritime provinces and in the far north of Canada.

**Figure 3:**
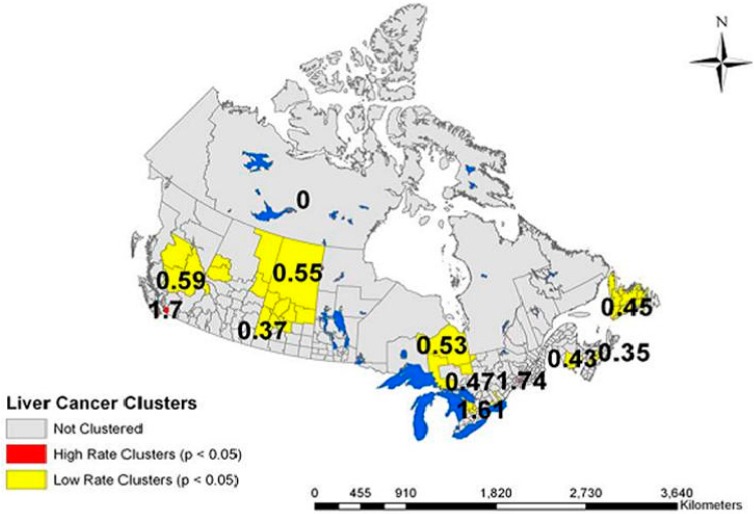
Cluster identification of liver cancer cases in Canada between 1996 and 2004, using spatial scan statistic (Satscan) software. The figure depicts regions of high and low rate clusters.

The spatial scan statistic confirmed that the high rate clusters of liver cancer were statistically significant (*p* < 0.05) in the urban centers of Vancouver (rate ratio (RR) = 1.7), Montreal (RR = 1.74) and Toronto (RR = 1.6), but not Calgary (Figure 3). Statistically significant low rate clusters were identified in north-central British Columbia (RR = 0.59), north-central Saskatchewan (RR = 0.55), northeastern Ontario (RR = 0.53), areas of southern Ontario (RR = 0.47) and the Maritime provinces (RR = 0.45, 0.35 and 0.43).

**Figure 4:**
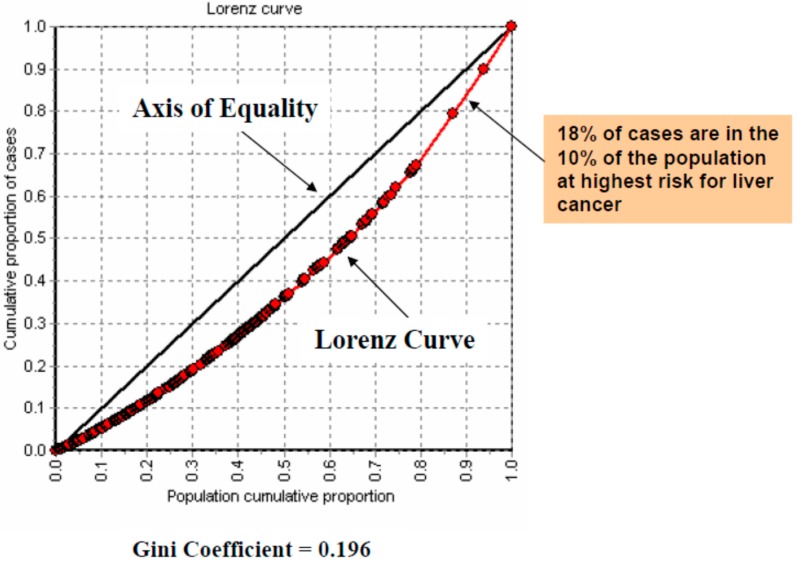
The Gini coefficient describes the degree to which cases are distributed equally in relationship to the population at risk.

The Gini coefficient for liver cancer incidence from 1996–2004 was 0.196 (Figure 4), suggesting a modest level of inequality in liver cancer incidence across geographic areas in Canada. The Lorenz curve used to calculate the Gini coefficient plots the ranked cumulative proportion of liver cancer incident cases against the ranked cumulative proportion of the population at risk. The minimal deflection of the Lorenz curve downwards from the axis suggests that liver cancer cases are not highly spatially concentrated in a small number of geographic areas in Canada; rather that liver cancer cases are spread out evenly in proportion to the population at risk. Reading directly from the Lorenz curve, the 10% of the population living in those areas of Canada having the highest liver cancer incidence rates contain 18% of the liver cancer cases.

As shown in Table 2, the results of the negative binomial regression analysis in a partially adjusted model (variables adjusted only for age and gender) revealed that none of the surrogate markers of cyanobacterial toxin exposure (% land devoted to agriculture, cattle and swine density) are associated with an increased RR for liver cancer. In terms of known risk factors (Table 3), liver cancer rates were significantly higher in the expected direction for Hepatitis B viral infection (RR = 1.16, *p* < 0.0014), recent immigrant status (RR = 1.89, *p* < 0.0001) and urban residence (RR = 1.25, *p* < 0.0001). However, an unexpectedly low rate was observed for the highest alcohol abuse tertile (RR = 0.84, *p* < 0.0321), and Hepatitis C viral infection was not associated with an increased RR. Consistent with the smoothed thematic map of liver cancer incidence, significantly elevated RR were observed for the Northwest Territories, British Columbia, Alberta and Manitoba as compared to Newfoundland, which had the lowest liver cancer incidence rate. Statistically significant RRs were not observed for any of the other predictive variables.

**Table 2:** Univariant Analysis Assessing the Impact of Cyanobacterial Surrogate Markers on Liver Cancer Development within Canada.

Variable	Range	Overall RR (95% CI-RR)
Agriculture	>62.68	1.00
(%)	23.66–62.68	1.05 (0.92–1.20)
2006	<23.66	1.11 (0.98–1.25)
Cattle Density	<62,460	1.00
Animals/1000 km^2^	62,460–262,169	0.96 (0.74–1.01)
2006	>262,169	1.27 (0.95–1.70) **
Swine Density	<33.67	1.00
Animals/1000 km^2^	33.67–142,937	0.93 (0.82–1.05)
2006	>142,937	1.00 (0.82–1.23)

Note: Relative risk (RR) reference value of 1.00 determined using Jenks Natural Breaks algorithm. ** *p* < 0.01.

**Table 3:** Univariant Analysis Assessing the Impact of Socio-Demographic Factors on Liver Cancer Development within Canada.

Variable	Range	Overall RR (95% CI-RR)
Hepatitis B (Cases/100 000), 1989–2004	<3.55	1.00
	3.55–5.24	0.72 (0.61–0.65) **
	>5.24	1.16 (1.06–1.27) **
Hepatitis C (Cases/100 000), 1989–2004	<36.21	1.00
	36.21–56.49	0.94 (0.85–1.05)
	>56.49	0.99 (0.87–1.27)
Alcohol Abuse (%), 40–64 yrs, 2007	<13.5	1.00
	13.5–18.76	1.00 (0.89–1.13)
	>18.76	0.84 (0.72–0.99) *
Recent Immigration (%), 2001	<1.28	1.00
	1.28–4.42	1.18 (1.05–1.33) **
	>4.42	1.89 (1.58–2.27) **
Urban/Rural	Rural	1.00
	Urban	1.25 (1.12–1.40) **
Province	10 Newfoundland	1.00
	24 Quebec	n/s
	45 Manitoba	1.73 (1.29–2.50) **
	48 Alberta	1.60 (1.31–2.49) **
	59 British Columbia	1.83 (1.33–2.57) **
	63 Nunavut	2.22 (1.02–4.62) *

Note: Relative risk (RR) reference value of 1.00 determined using Jenks Natural Breaks algorithm. * *p* < 0.05; ** *p* < 0.01.

## 4. Discussion

The results of this study reveal that liver cancer rates are not higher in regions of the country where cyanobacterial contamination of the lakes and drinking water would be expected to be most problematic; as reflected by surrogate markers for cyanotoxin exposure (agriculture, cattle and swine density). The results also indicate that as in other parts of the world, liver cancers in Canada tend to be more common in those individuals with chronic hepatitis B infections, areas where there has been significant recent immigration and residence within large urban centres.

Further supporting the finding that cyanobacterial contamination of lakes and drinking water does not represent a significant risk for liver cancer development in Canada, are the results of the Gini coefficient analysis. The relatively low coefficient suggests that liver cancer cases are not highly concentrated in a small number of geographic areas of the country (as might be expected with cyanobacterial contamination of drinking water), but rather, are distributed across all population groupings. This suggests that single point sources of exposure leading to liver cancer are unlikely in discrete geographic populations, and the causative conditions are more widely distributed.

Overall, the findings of this study differ from what was described by investigators in China, Serbia and the United States. Specifically in China, Yu *et al.* reported the incidence of liver cancer to be highest in rural populations receiving drinking water from cyanobacterial contaminated pond and ditch sources, compared to those whose drinking water was derived from presumed cyanobacterial-free deep wells or rivers [34,47]. Similarly, in Serbia, Scircev *et al.* [35] reported 1.5–4.4 fold increases in primary liver cancer (PLC) mortality rates in regions of Central Serbia receiving their drinking water from cyanobacterial contaminated surface water reservoirs, compared to regions receiving their drinking water from non-cyanobacterial contaminated underground sources. Finally, in the state of Florida, Fleming *et al.* [36] documented a 1.38 fold increase risk of liver cancer in communities receiving drinking water from cyanobacterial contaminated surface water reservoirs, compared to non-exposed contiguous matched control zones.

There are a number of potential explanations for why the results of the present study differ from those referred to above. First, our study population consisted of the entire national Canadian population rather than a smaller regional or state sub-population. Thus, relevant local level relationships are more likely to have been undetected in our study. Second, our limited demographic data meant that age and gender could only be controlled for at the ecological level. To partially overcome this barrier, analyses were restricted to the 40 plus population in whom the majority of liver cancer cases occur. Third, liver cancer case data received from the National Cancer Registry were highly suppressed (92 CDs with suppressed data) and as a result, the geographic trends observed may represent an artifact of the data imputation method used to overcome data suppression. To minimize the impact of this limitation, spatial smoothing and cluster identification techniques were employed. Fourth, the liver cancer case data received from the National Cancer Registry was aggregated to Census Divisions (CDs). CDs are highly variable in size, with some containing populations in excess of 2.5 million people. As a result, an examination of the variation in liver cancer rates within large urban centers was not feasible, resulting in a likely truncation of observed liver cancer variability across Canada. Furthermore, the ecological regression models used in the study were often imprecise because they had to classify all individuals living in a geographic area with the same ecological predictor value. This may not have been a problem for ecological modeling of predictors which homogenously affected the whole population in a CD (e.g., urban residence), but was likely a significant problem for predictors where there may have been significant heterogeneity within geographic areas (e.g., family income in large urban CDs). Thus, the modeling results may have been biased in some cases, with the observed rate ratios either muted or possibly in a direction opposite to what one would have observed if modeling could have occurred at a finer scale. Fifth, for a number of predictor variables, the data was not available at the CD level and had to be spatially interpolated from another scale. For example, alcohol abuse had to be interpolated from the regional health authority area, and hepatitis B and C infections were interpolated from scale of the province. In the case of hepatitis B and C, it is unlikely that all CDs in a province had the same rates of these infections. In addition, the definition of urban/rural used in the study was potentially problematic in that many CDs are of sufficient size that they often contain both significant urban and rural populations. Sixth, we utilized surrogate markers of cyanobacterial contamination to document sources of contaminated drinking water, rather than the direct cyanotoxin or cyanobacterial quantification used by Chinese, Serbian and American investigators. Hence, the lack of Canadian wide cyanobloom or MC toxin concentration data and inability to find more sensitive upstream proxies of exposure may have compromised our results. Finally, it should be noted that exposure to other carcinogens or co-carcinogens such as aflatoxins and hepatitis B viral infections may have contributed to and perhaps, even been responsible for the apparent association between cyanotoxin exposure and liver cancer rates reported in the previous studies.

## 5. Conclusions

The data generated in this study suggest that cyanotoxin exposure does not appear to be contributing to the increasing incidence of liver cancer in Canada. However, the results do underscore the need for further research at the provincial level (monitoring of cyanobacterial or cyanotoxin contamination of lakes and drinking water sources) to overcome the limitations encountered at the national level. Future studies in this area should be conducted using unsuppressed national level data, which is currently prohibited by release guidelines, in order to facilitate a more robust analysis of the epidemiology of liver cancer in Canada.
